# ILD-GAP combined with the monocyte ratio could be a better prognostic prediction model than ILD-GAP in patients with interstitial lung diseases

**DOI:** 10.1186/s12890-023-02833-6

**Published:** 2024-01-05

**Authors:** Momo Hirata, Yu Hara, Hiroaki Fujii, Kota Murohashi, Yusuke Saigusa, Shiqi Zhao, Miyu Kobayashi, Ryo Nagasawa, Yoichi Tagami, Ami Izawa, Yukiko Otsu, Keisuke Watanabe, Nobuyuki Horita, Nobuaki Kobayashi, Takeshi Kaneko

**Affiliations:** 1 Department of Pulmonology, Yokohama City University Graduate School of Medicine, 3-9 Fukuura, Kanazawa-ku, Yokohama, Kanagawa 236-0004 Japan; 2 Department of Biostatistics, Yokohama City University Graduate School of Medicine, Yokohama, Japan

**Keywords:** Composite scoring system, Diffusion capacity of lung for carbon monoxide, Idiopathic pulmonary fibrosis, Interstitial lung disease, Monocyte ratio

## Abstract

**Background:**

The ILD-GAP scoring system is known to be useful in predicting prognosis in patients with interstitial lung disease (ILD). An elevated monocyte count was associated with increased risks of IPF poor prognosis. We examined whether the ILD-GAP scoring system combined with the monocyte ratio (ILD-GAPM) is superior to the conventional ILD-GAP model in predicting ILD prognosis.

**Methods:**

In patients with ILD treated between April 2013 and April 2017, we were retrospectively assessed the relationships between baseline clinical parameters, including age, sex, Charlson Comorbidity Index score (CCIS), ILD diagnosis, blood biomarkers, pulmonary function test results, and disease outcomes. In ILD patients were included idiopathic pulmonary fibrosis (IPF), idiopathic nonspecific interstitial pneumonia (iNSIP), collagen vascular disease-related interstitial pneumonia (CVD-IP), chronic hypersensitivity pneumonitis (CHP), and unclassifiable ILD (UC-ILD). We also assessed the ability to predict prognosis was compared between the ILD-GAP and ILD-GAPM models.

**Results:**

A total of 179 patients (mean age, 73 years) were assessed. All of them were taken pulmonary function test, including percentage predicted diffusion capacity for carbon monoxide. ILD patients included 56 IPF cases, 112 iNSIP and CVD-IP cases, 6 CHP cases and 5 UC-ILD cases. ILD-GAPM provided a greater area under the receiver-operating characteristic curve (0.747) than ILD-GAP (0.710) for predicting 3-year ILD-related events. Furthermore, the log-rank test showed that the Kaplan-Meier curves in ILD-GAPM were significantly different by stage (*P* = 0.015), but not by stage in ILD-GAP (*P* = 0.074).

**Conclusions:**

The ILD-GAPM model may be a more accurate predictor of prognosis for ILD patients than the ILD-GAP model.

**Supplementary Information:**

The online version contains supplementary material available at 10.1186/s12890-023-02833-6.

## Background

Interstitial lung disease (ILD) is characterized by alveolitis resulting in progressive fibrosis. It is classified by various radiological, pathological, and morphological patterns, including usual interstitial pneumonia (UIP), nonspecific interstitial pneumonia (NSIP), organized pneumonia, respiratory bronchiolitis, desquamative interstitial pneumonia, diffuse alveolar damage, and combinations thereof. The clinical course and rate of progression of ILDs are extremely variable from patient to patient [1]. Especially, among patients with idiopathic pulmonary fibrosis (IPF), genetic factors (sporadic or familial) could play a pivotal role in the risk of disease progression [2]. Although official statements from the American Thoracic Society/European Respiratory Society/Japanese Respiratory Society/Latin American Thoracic Association (ATS/ERS/JRS/ALAT) indicate that clinical parameters such as clinical presentation, lung function, the distance or desaturation during 6-minute-walk test, and UIP extent on high-resolution computed tomography are associated with an increased risk of death, no clinical parameters have been established to accurately predict the prognosis for ILD in its various stages [3, 4]. In patients with ILD, the combination of various kind or parameters such as peripheral blood biomarkers, physiological and radiological measurements, and functional ability and supplemental oxygen requirement has been reported to provide more accurate prognostic information [5–10]. The GAP index proposed by Ley et al. shows that four parameters, gender (G), age (A), predicted forced ventilation volume (%FVC), and carbon monoxide diffusing capacity (%D_Lco_) (P), can be used to predict mortality in patients with IPF [5]. By adding the conditions of chronic ILD subtypes such as IPF, idiopathic NSIP (iNSIP), collagen vascular disease-related interstitial pneumonia disease (CVD-IP), chronic hypersensitivity pneumonia (CHP), and unclassifiable ILD (UC-ILD) to the conventional GAP model, the ILD-GAP has been reported to predict mortality in the major chronic ILD subtypes as ILD-GAP [6]. Yagyu et al. reported that the composite scoring system accounting for IPF diagnosis, comorbidity, and %D_Lco_ could provide a useful tool for predicting 3-year prognosis in patients with ILD [9].

One of the most important mechanisms of IPF progression is the alveolar type II epithelial dysfunction which is mainly caused by aging, environmental factors, and genetic determinants, however, there was no established biomarker for prediction of disease progression for patients with IPF [11]. Several inflammation indices, such as the monocyte count (normal range is 200–600/μL or 2–10%), neutrophil-to-lymphocyte ratio (NLR), platelet-to-lymphocyte ratio (PLR), systemic inflammation index (SII), systemic inflammation response index (SIRI), and aggregate index of systemic inflammation (AISI), were found to be associated with IPF development and prognosis [12]. In addition, from the retrospective pooled analysis of four phase III, randomized, placebo-controlled trials [ASCEND (NCT01366209), CAPACITY (NCT00287729 and NCT00287716), and INSPIRE (NCT00075998)], an elevated monocyte count was associated with increased risks of IPF progression, hospitalization, and mortality [13]. The monocyte count provides a simple and inexpensive method of obtaining prognostic information and can be measured repeatedly; it is thus expected to be a biomarker that can be used frequently in clinical practice.

The present retrospective study investigated the accuracy of the ILD-GAP scoring system combined with the blood monocyte ratio (ILD-GAPM) for predicting the prognosis of patients with ILD.

## Methods

### Study location and enrolled patients

**Fig. 1:**
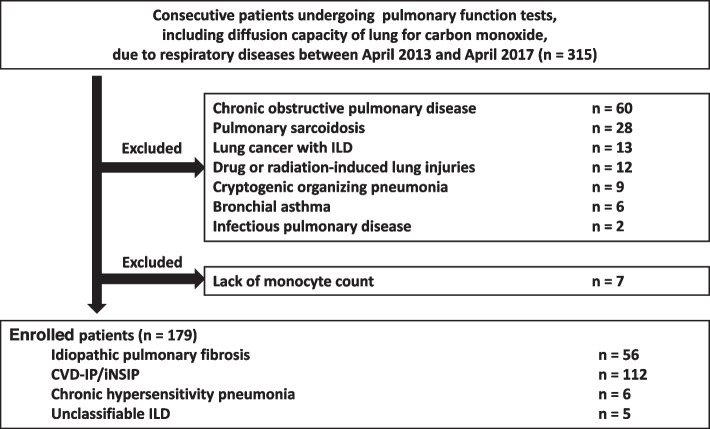
Flowchart of the participant selection process. Abbreviations: CVD-IP, collagen vascular disease-related interstitial pneumonia; ILD, interstitial lung disease; iNSIP, idiopathic NSIP

This retrospective, observational study was performed using data from patients treated at Yokohama City University Hospital between April 2013 and April 2017. The medical records of all patients with ILD who met the following inclusion criteria were reviewed: patients with IPF, iNSIP, CVD-IP, CHP, and UC-ILD in stable condition who were able to perform pulmonary function tests including D_Lco_. ILD patients in stable condition were defined as patients who had not experienced acute respiratory worsening such as an acute exacerbation (AE), infection, pulmonary embolism, pneumothorax, or pulmonary oedema until pulmonary function testing [14]. As shown in Fig. 1, pulmonary sarcoidosis, lung cancer with ILD at the time of enrolment, cryptogenic organizing pneumonia, drug or radiation-induced lung injuries, chronic obstructive pulmonary disease, bronchial asthma, and infectious pulmonary disease were excluded. An AE was defined as significant respiratory deterioration including clinical worsening of dyspnea, hypoxemia, or the worsening or severe impairment of gas exchange characterized by new bilateral ground-glass opacification/consolidation superimposed on a background pattern consistent with IPF pattern not fully explained by cardiac failure or fluid overload [15]. A diagnosis of idiopathic interstitial pneumonia (IIP) was confirmed by physical findings, serological testing, findings from high-resolution computed tomography (HRCT), and lung biopsy specimens, based on the official statement for IIP and patients from whom a lung biopsy could not be obtained were diagnosed based on the radiological classification [1, 2, 4]. The diagnosis of CVD-IP was confirmed by physical findings, serological testing, and HRCT findings consistent with ILD. CHP was diagnosed based on previously established criteria [16]. The present study was conducted according to transparent reporting of a multivariable prediction model for individual prognosis or diagnosis (TRIPOD) statement (Supplement file).

### Data collection

The relationships between baseline clinical parameters including age, sex, Charlson Comorbidity Index score (CCIS), ILD diagnosis, blood biomarkers, pulmonary function test results, HRCT scores, and the disease outcome were evaluated. The CCIS is an established and widely used tool to measure comorbidity burden with 19 different medical conditions [17]. It is scored based on the number and severity of comorbidities, with a higher CCI indicating greater comorbidity burden and severity. It was developed to assess the risk of death from comorbidities and has been widely used as a prognostic indicator for patients with colorectal cancer, advanced non-small cell lung carcinoma, and acute myocardial infarction [18–20]. The disease outcome is 3-year ILD-related events included ILD-related mortality such as respiratory failure and first AE within 3 years after pulmonary function testing. For patients who did not die in our hospital, the disease outcomes were confirmed by telephone. In addition, only one patient (0.5%), who was transferred to another hospital for best supportive case due to severe deterioration of respiratory status, was lost to follow-up; therefore, the transfer date of that patient was selected as the decision date for disease outcome.

### HRCT scoring

The HRCT findings were evaluated using the semiquantitative scoring method described by Ooi et al. [21]. The lungs were divided into six distinct zones, three on each side. Ground-glass opacity (GGO) and honeycombing on HRCT were then scored based on the percentage of disease extent in each of the 6 lung lobes. A global score was calculated by adding the scores for each abnormality in all lobes. HRCT was performed at the diagnosis of ILDs; each scan was independently assessed by three pulmonologists (experience-year: more than 10 years)).

### Details of the ILD-GAP and ILD-GAPM classifications

The ILD-GAP model was developed for application across all ILD subtypes including iNSIP, CVD-IP, CHP, and UC-ILD to provide cause-specific survival estimates using a single risk prediction model including the original GAP model for IPF patients that accounted for better adjusted survival in these patients [4]. As shown in Table 1, the predictor variables considered in this model include gender, age, lung physiology variables (%FVC and %D_Lco_), and these ILD subtypes. The ILD-GAP score was calculated by subtracting 2 points from the existing GAP score for patients with a diagnosis of iNSIP, CVD-IP, or CHP and the discrimination and calibration of the ILD-GAP score were tested in each ILD subtype with the concordance index (C-index) calculated using the bootstrap method [6]. The ILD-GAP score was calculated by combining points assigned to these variables to obtain a total point score, and they are then divided into stage I (≤ 1 point), stage II (2, 3 points), stage III (4, 5 points), and stage IV (> 5 points). Also, the enrolled patients could be divided into the low monocyte ratio group (≤ 8.5 points) and the high monocyte ratio group (> 8.5 points), because the median value of monocyte ratio from our data was 8.5 points. The ILD-GAPM score was calculated by combining points assigned to the variables from the ILD-GAP model and the monocyte ratio to obtain a total point score, and they are then divided into stage I (≤ 1 point), stage II (2, 3 points), stage III (4, 5 points), and stage IV (> 5 points).

**Table 1:** Details of the ILD-GAP and ILD-GAPM models

		ILD-GAP model	ILD-GAPM model
		Points	Points
**ILD diagnosis**	**IPF / UC-ILD**	0	0
	**CVD-IP / iNSIP /CHP**	−2	− 2
**Sex**	**Female**	0	0
	**Male**	1	1
**Age, y**	**≤60**	0	0
	**61–65**	1	1
	**> 65**	2	2
**%FVC**	**> 75**	0	0
	**50–75**	1	1
	**< 50**	2	2
**%D**_**Lco**_	**> 55**	0	0
	**36–55**	1	1
	**≤35**	2	2
	**Cannot perform**	3	3
**%Monocytes**	**≤8.5**		0
	**> 8.5**		1

Abbreviations: *CHP* chronic hypersensitivity pneumonitis, *CVD-IP* collagen vascular disease-related interstitial pneumonia, *GAP* gender/age/physiology, *GAPM* gender/age/physiology/blood monocyte ratio, *ILD* interstitial lung disease, *iNSIP* idiopathic nonspecific interstitial pneumonia, *IPF* idiopathic pulmonary fibrosis, *%D*_*Lco*_ percentage predicted diffusion capacity of lung for carbon monoxide, *%FVC* percentage predicted forced vital capacity, *UC-ILD* unclassifiable interstitial lung disease

### Statistical analysis

Statistical data were analyzed using JMP16 (SAS Institute Inc., North Carolina, USA) and R software, version 3.5.1 (The R Foundation for Statistical Computing, Vienna, Austria). The results were expressed as mean ± standard deviation. Chi-square and Wilcoxon rank-sum tests were used for between-group comparisons. Univariate analyses were used to identify the main predictors of 3-year ILD-related events. The area under the time-dependent receiver-operating characteristic curve (ROC) analysis (AUC), C-index, and Akaike’s information criterion (AIC) were used to evaluate the prognostic predictive performances of the ILD-GAP and ILD-GAPM scoring systems. The confidence intervals (CI) for time-dependent AUC and C-index were calculated using the bootstrap method based on normal approximation of the statistics. The number of resampling to estimate the variances was set to 2000. Kaplan-Meier curves were used to compare 3-year ILD-related events and 3-year all-cause mortality between groups by a scoring system, and log-rank tests were performed with stratification based on identified predictors; values of *P* <  0.05 were considered significant.

## Results

### Patients’ characteristics

Table 2 shows the clinical characteristics of the 179 patients evaluated, including IPF in 56 cases, iNSIP and CVD-IP in 112 cases, CHP in 6 cases, and UC-ILD in 5 cases. CVD-IP included rheumatoid arthritis, anti-neutrophil cytoplasmic antibody-associated vasculitis, polymyositis/dermatomyositis, and Sjögren’s syndrome. Especially in the IPF group, the incidence of males was high, and %D_Lco_ was the lowest. The ILD-GAP scores of IPF and UC-ILD were similar and higher than those of the other ILDs. Anti-fibrotic agents were used in 10 (9 IPF and 1 iNSIP) patients. Anti-inflammatory agents including corticosteroids or immunosuppressants were used mainly in the patients with CVD-IP or iNSIP.

**Table 2:** Patients’ characteristics

Characteristics	Overall cases	IPF	CVD-IP/iNSIP	CHP	UC-ILD	*P* value
**Total number, n (%)**	179 (100)	56 (31)	112 (63)	6 (3)	5 (3)	
**Age, y**	73 ± 9	73 ± 7	71 ± 9	68 ± 21	72 ± 4	0.637
**Male sex, n (%)**	122 (68)	49 (88)	64 (57)	4 (67)	5 (100)	< 0.001
**Blood biomarkers**						
**LDH, IU/L**	218 ± 73.7	211.6 ± 54.9	222.1 ± 83.2	243.8 ± 58.2	188.2 ± 25.4	0.181
**SP-D, ng/mL**	197.9 ± 240.1	170.1 ± 140.9	208.2 ± 259.7	377.7 ± 517.4	63.5 ± 27.2	0.097
**KL-6, U/mL**	843.1 ± 1090.4	727.4 ± 435.1	808.4 ± 1030.8	2692 ± 3265.3	405.3 ± 154.4	0.118
**Monocyte ratio, %**	8.6 ± 2.8	8.3 ± 2.2	8.7 ± 2.8	10.0 ± 5.9	7.3 ± 2.2	0.701
**Monocyte count, /μL**	573.9 ± 226.1	627.0 ± 250.5	555.3 ± 211.8	537.8 ± 170.3	437.7 ± 237.0	0.290
**Pulmonary function tests**						
**FVC, %predicted**	94.2 ± 18.8	93.4 ± 18.7	94.2 ± 19.3	92.8 ± 10.8	105 ± 19.6	0.397
**D**_**Lco**_**, %predicted**	92.9 ± 30.5	81.2 ± 27.9	98.2 ± 30.6	83.1 ± 17.6	118.9 ± 25.0	0.001
**ILD-GAP score**	1.4 ± 1.4	3 ± 0.9	0.6 ± 0.7	0.7 ± 0.5	3.2 ± 0.4	< 0.001
**HRCT score**						
**Honeycomb score, points**	0.7 ± 1.8	1.8 ± 2.7	0.2 ± 0.8	0.3 ± 0.8	0	< 0.001
**GGO score, points**	4.7 ± 2.9	5.2 ± 2.5	4.4 ± 2.5	8.2 ± 8.2	3.0 ± 1.0	0.064
**Treatment**						
**Anti-fibrotic agents, n (%)**	10 (5)	9 (16)	1 (1)	0 (0)	0 (0)	0.001
**Corticosteroid, n (%)**	39 (22)	9 (16)	29 (26)	1 (17)	0 (0)	0.300
**Immunosuppressant, n (%)**	21 (12)	1 (2)	20 (18)	0 (0)	0 (0)	0.003
**Outcome**						
**Follow-up, days**	679 ± 337	652 ± 329	697 ± 347	775 ± 294	484 ± 211	0.410
**3-y ILD-related events, n (%)**	21 (12)	10 (18)	10 (9)	0 (0)	1 (20)	0.261

Serum SP-D could be measured in 122 patients (68%)

3-y ILD-related events include cause-specific mortality due to ILD, and first AE within 3 years after pulmonary function testing

Abbreviations: *AE* acute exacerbation, *CHP* chronic hypersensitivity pneumonia, *CVD-IP* collagen vascular disease-related interstitial pneumonia, *GAP* gender/age/physiology, *GGO* ground-grass opacity, *HRCT* high-resolution computed tomography, *ILD* interstitial lung disease, *IPF* idiopathic pulmonary fibrosis, *KL-6* Krebs von den Lungen-6, *LDH* lactate dehydrogenase, *%D*_*Lco*_ percentage predicted diffusion capacity of lung for carbon monoxide, *%FVC* percentage predicted forced vital capacity, *SP-D* surfactant protein-D, *UC-ILD* unclassifiable interstitial lung disease

### Univariate analysis of primary predictors of 3-year ILD-related events

**Fig. 2:**
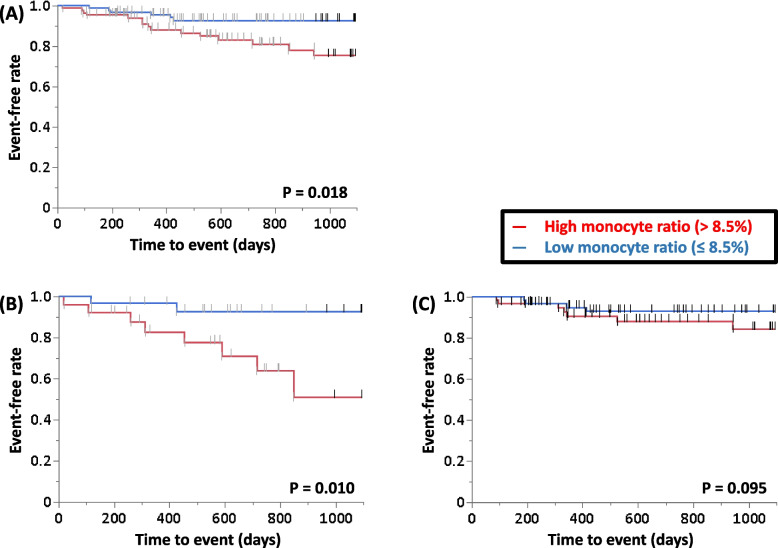
Survival curves of the patients with IPF and non-IPF according to peripheral blood monocyte ratio. **A** All patients (*N* = 179); (**B**) IPF patients (*N* = 56); (**C**) Non-IPF patients (*N* = 123). Abbrevialtions: IPF, idiopathic pulmonary fibrosis

**Fig. 3:**
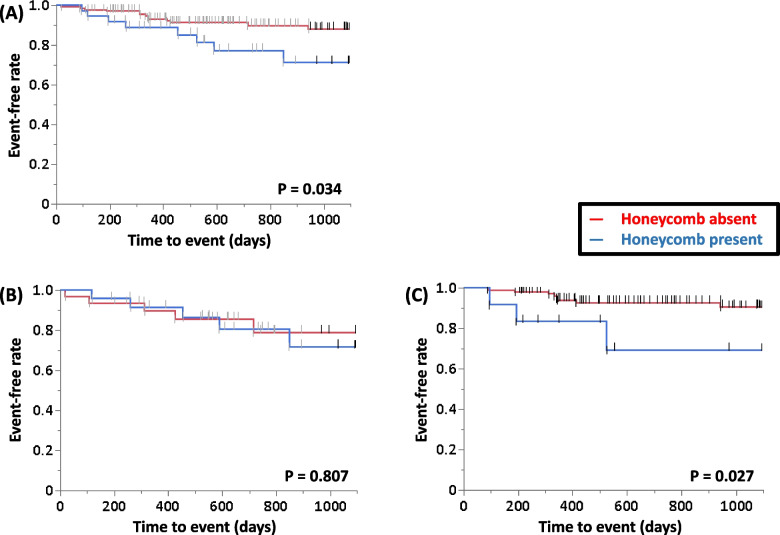
Survival curves of the patients with IPF and non-IPF with or without honeycomb. **A** All patients (*N* = 179); (**B**) IPF patients (*N* = 56); (**C**) Non-IPF patients (*N* = 123). Abbrevialtions: IPF, idiopathic pulmonary fibrosis

ROC curve analysis of the ability of the monocyte ratio and count to predict 3-year ILD-related events for all patients was performed, and the AUC of the monocyte ratio was relatively higher than that of the monocyte count (0.62 vs. 0.60). Therefore, to determine the primary predictors of 3-year ILD-related events, univariate analysis was performed in the patients with IPF or non-IPF using the following parameters: age, sex, CCIS, monocyte ratio, HRCT scores, ILD-GAP score, %FVC, and %D_Lco_ (Table 3). These showed that the monocyte ratio was a significant predictor of 3-year ILD-related events in the patients with IPF and the ILD-GAP score was significant in the patients with non-IPF. Table 4 shows the clinical characteristics of IPF or non-IPF with high (> 8.5 points) or low (≤ 8.5 points) monocyte ratio. With reference to the value of the monocyte ratio for all patients, survival curves were compared between patients in the high and low monocyte ratio groups, and significant differences were found by the log-rank test for all enrolled patients and in patients with IPF (*P* = 0.018, Fig. 2A; and *P* = 0.010, Fig. 2B, respectively). A similar tendency was observed in patients with non-IPF (*P* = 0.095, Fig. 2C). Survival curves were compared between patients in the honeycomb present and absent groups, and significant difference was found by the log-rank test for all enrolled patients and in patients with non-IPF, but not in those with IPF (*P* = 0.034, Fig. 3A; *P* = 0.027, Fig. 3C; and *P* = 0.807, Fig. 3B, respectively).

**Table 3:** Univariate analysis of primary predictors of 3-y ILD-related events

**IPF group**			
**Variable**	**Hazard ratio**	**95% CI**	**P**
**Age**	1.025	0.915–1.154	0.669
**Sex (Male)**	1.464	0.128–16.780	0.754
**Monocyte ratio**	1.466	1.027–2.206	0.035
**Honeycomb score**	1.078	0.741–1.491	0.663
**GGO score**	0.994	0.710–1.349	0.971
**ILD-GAP score**	0.757	0.241–1.970	0.583
**%FVC**	0.972	0.925–1.015	0.206
**%D**_**Lco**_	0.991	0.954–1.022	0.590
**Non-IPF group**			
**Variable**	**Hazard ratio**	**95% CI**	**P**
**Age**	0.978	0.900–1.074	0.617
**Sex (Male)**	0.337	0.067–1.707	0.183
**Monocyte ratio**	1.063	0.861–1.266	0.538
**Honeycomb score**	1.221	0.657–1.844	0.449
**GGO score**	0.953	0.713–1.189	0.713
**ILD-GAP score**	2.130	1.044–4.174	0.038
**%FVC**	0.990	0.954–1.026	0.587
**%D**_**Lco**_	1.000	0.972–1.026	0.990

Abbreviations: *CI* confidence interval, *GAP* gender/age/physiology, *GGO* ground-glass opacity, *ILD* interstitial lung disease, *IPF* idiopathic pulmonary fibrosis, *%D*_*Lco*_ percentage predicted diffusion capacity of lung for carbon monoxide, *%FVC* percentage predicted forced vital capacity

**Table 4:** Comparison of the clinical parameters between the patients with high and low monocyte ratio

Variable	IPF with high monocyte ratio	IPF with low monocyte ratio	Non-IPF with high monocyte ratio	Non-IPF with low monocyte ratio	*P* values
**Total number, n (%)**	26 (14)	30 (17)	62 (35)	61 (34)	
**Age, y**	74 ± 6	72 ± 8	72 ± 10	71 ± 10	0.365
**Male sex, n (%)**	24 (92)	25 (83)	38 (61)	35 (57)	0.002
**Blood biomarkers**					
**LDH, IU/L**	221.5 ± 63.1	203.1 ± 46.8	234.6 ± 97.4	208.8 ± 57.4	0.248
**SP-D, ng/mL**	147.9 ± 127.3	186.6 ± 150.8	180.0 ± 231.9	240.0 ± 310.1	0.503
**KL-6, U/mL**	689.5 ± 523.9	761.0 ± 344.6	918.5 ± 1185.4	877.2 ± 1384.8	0.130
**Monocyte ratio, %**	10.2 ± 1.2	6.6 ± 1.3	10.8 ± 2.8	6.7 ± 1.5	< 0.001
**Monocyte count, /μL**	721.6 ± 283.4	545.1 ± 186.4	646.8 ± 206.5	451.0 ± 165.1	< 0.001
**HRCT scores**					
**Honeycomb score, points**	0.9 ± 1.7	2.5 ± 3.2	0.2 ± 0.6	0.3 ± 1.0	< 0.001
**GGO score, points**	4.3 ± 2.6	5.9 ± 2.2	4.8 ± 2.5	4.2 ± 3.5	0.001
**ILD-GAP score**	4.2 ± 0.8	3.8 ± 0.9	0.5 ± 1.0	0.6 ± 1.2	< 0.001
**ILD-GAPM score**	5.2 ± 0.8	3.8 ± 0.9	1.5 ± 1.0	0.6 ± 1.2	< 0.001
**Pulmonary function tests**					
**%FVC, %predicted**	93.8 ± 20.7	93.2 ± 17.1	97.5 ± 18.0	91.6 ± 19.6	0.277
**%D**_**Lco**_**, %predicted**	80.3 ± 32.4	82.0 ± 23.8	98.8 ± 33.3	97.7 ± 27.0	0.007

Serum SP-D could be measured in 122 patients (68%)

Abbreviations: *GAP* gender/age/physiology, *GAPM* gender/age/physiology/blood monocyte ratio, *GGO* ground-glass opacity, *HRCT* high-resolution computed tomography, *ILD* interstitial lung disease, *IPF* idiopathic pulmonary fibrosis, *KL-6* Krebs von den Lungen-6, *LDH* lactate dehydrogenase, *%D*_*Lco*_ percentage predicted diffusion capacity of lung for carbon monoxide, *%FVC* percentage predicted forced vital capacity, *SP-D* surfactant protein-D

### Comparison of the survival curves between the ILD-GAP and ILD-GAPM models

**Fig. 4:**
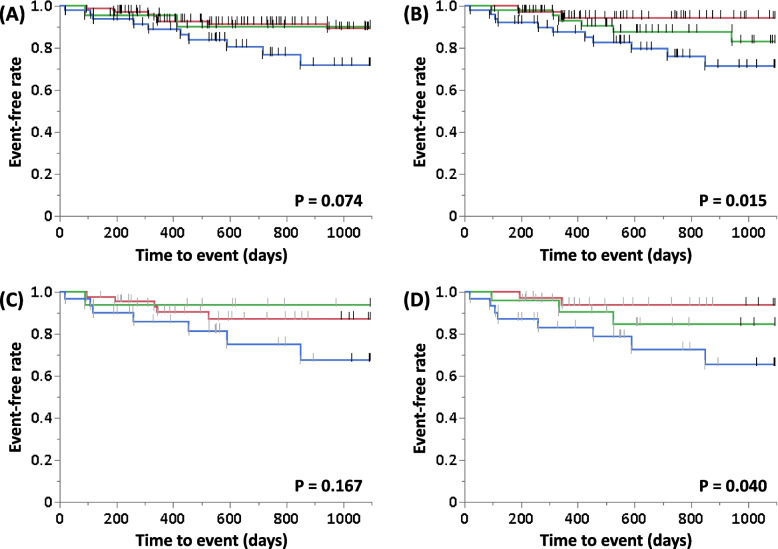
Survival curves of ILD-GAP and ILD-GAPM models. **A** ILD-GAP model; (**B**) ILD-GAPM model; (**C**) ILD-GAP model for the patients with < 90% D_Lco_; (**D**) ILD-GAP model for the patients with < 90% D_Lco_ (red line, ILD-GAPM stage I; green line, stage II; blue line, stage ≥III). Abbrevialtions: GAP, gender / age / physiology; GAPM, gender / age / physiology / blood monocyte ratio; ILD, interstitial lung disease

In Fig. 4, Kaplan-Meier curves for 3-year ILD-related events are compared by stage using the original ILD-GAP model classification; when compared by ILD-GAP stage, the log-rank test showed no significant difference between these groups (Fig. 4A; *P* = 0.074). On the other hand, when these curves were compared by ILD-GAPM stage, the log-rank test showed that the survival curves for these groups were significantly different (Fig. 4B; *P* = 0.015). Furthermore, we also compared survival curves between the ILD-GAP and ILD-GAPM models in the group with lower D_Lco_ (targeted at 90% or less, which is the median D_Lco_ among all enrolled patients) and found that the same tendency as above were observed. (Fig. 4C and D).

### Accuracy of composite scoring models in predicting 3-year ILD-related events

Time-dependent AUC, C-index values, and AIC values for the ILD-GAP and ILD-GAPM models were calculated to compare the accuracy of each model for ILD-related events over a 3-year period. The AUCs, C-index values, and AIC values all indicated greater accuracy with the ILD-GAPM model (AUC, 0.747; C-index, 0.630; AIC, 198.1) than with the ILD-GAP model (AUC, 0.710; C-index, 0.556; AIC, 201.2) (Table 5).

**Table 5:** Predictability of ILD-related events by the ILD-GAP and ILD-GAPM models

	Time dependent AUC (95%CI)	C-index (95%CI)	AIC
**ILD-GAP model**	0.710 (0.584–0.832)	0.556 (0.449–0.663)	201.2
**ILD-GAPM model**	0.747 (0.623–0.871)	0.630 (0.522–0.737)	198.1

Abbreviations: *CI* confident intervals, *AIC* Akaike‘s information criterion, *AUC* areas under the time-dependent receiver-operating characteristic curve analysis, *GAP* gender/age/physiology, *GAPM* gender/age/physiology/blood monocyte ratio, *ILD* interstitial lung disease

## Discussion

The clinical course and rate of progression of ILD is extremely variable among patients due to various radiologic and pathologic morphologic patterns. No clinical parameters have been established to accurately predict the prognosis of ILDs with varying course. The ILD-GAP scoring system is widely used to predict the prognosis of ILD patients. In addition, the presence of elevated blood monocytes is reported to be associated with increased risks of IPF progression, hospitalization, and mortality in patients with IPF [13]. From the above, the combination of ILD-GAP and the blood monocyte ratio is estimated to be a highly versatile prognostic model in clinical practice, and in the present study the accuracy for predicting ILD prognosis of the ILD-GAPM model was investigated. As the results, our findings were that comparing survival curves between patients in the high (> 8.5 points) and low (≤ 8.5 points) monocyte ratio groups, significant differences were found by the log-rank test for all enrolled patients and in patients with IPF and the AUCs, C-index values, and AIC values for predicting 3-year ILD-related events all indicated greater accuracy with the ILD-GAPM model than with the ILD-GAP model.

Circulating monocytes have been reported to promote and predict IPF progression [13, 22, 23]. Scott et al., by performing cell deconvolution analysis of transcriptome data, reported an unexpected finding of an association between absolute and relative numbers of circulating monocytes and survival in patients with IPF [22]. To validate this finding, a retrospective pooled analysis of four phase III, randomized, placebo-controlled trials [ASCEND (NCT01366209), CAPACITY (NCT00287729 and NCT00287716), and INSPIRE (NCT00075998)] was performed by Kreuter et al., who demonstrated that an elevated monocyte count was associated with increased risks of IPF progression, hospitalization, and mortality [13]. Macrophages play key roles in all phases of adult wound healing, which are inflammation, proliferation, and remodeling. Circulating monocytes that migrate to the lung are differentiated uncommitted macrophages (M0), and they are broadly polarized to pro-inflammatory M1 macrophages and anti-inflammatory M2 macrophages [24, 25]. The interaction between M1 and M2 macrophages is reported to be closely correlated with disease progression in patients with ILD, including acute lung injury and IIPs, and having AEs or not [26–29]. In fact, we proposed that serum heme oxygenase (HO)-1 as a macrophage activation biomarker is useful for distinguishing between AE and stable ILD, and serum HO-1 levels were positively correlated with serum levels of SP-D and GGO score calculated from HRCT [30]. From the above, the mechanisms of immunocytological activation consisting of circulating monocytes, their migration to lung tissue, their differentiation to alveolar macrophages, and their activation could contribute to disease progression in patients with ILDs. Furthermore, the results of the present study suggest that the clinical impact of circulating monocytes on long-term prognosis may be particularly important in IPF.

The ILD-GAP model was reported to accurately predict mortality in major chronic ILD subtypes and at all stages of disease, but this predictability may depend on various background factors such as race, the severity of pulmonary function, and ILD subtypes [6, 31–35]. A retrospective analysis of a fibrotic HP (fHP) cohort in two Portuguese ILD centres showed that the ILD-GAP model is a good predictor of mortality in fHP, even after adjusting for other mortality risk factors in 141 fHP patients [31]. In another retrospective analysis of UC-ILD patients from the population-based ILD registry at Aarhus University Hospital, Denmark, the ILD-GAP was found to be the predictor of disease outcome [32]. On the other hand, from a retrospective, cross-sectional study of 179 participants with myositis-associated ILD in the Johns Hopkins Myositis Center database, the ILD-GAP risk prediction model was found to be a poor predictor of mortality in individuals [33]. Furthermore, in Japanese and Korean patients with IPF, it was concluded that it was difficult to accurately predict the prognosis of IPF, because there was no significant difference in survival between GAP stages II and III [34, 35]. In fact, in the present study we found that the ILD-GAP model provided less accurate information for predicting 3-year ILD-related events in patients with ILD than the ILD-GAPM model and there was also similar tendency among the patients with lower D_Lco_. Because fibrotic ILD requires early therapeutic intervention and accurate prognosis prediction in patients with relatively preserved pulmonary function is extremely important, the ILD-GAPM model may be prognostically advantageous in relatively early ILD patients, even though it would be important to validate these results in a multicentre, prospective cohort.

Although the ILD-GAPM model might have been shown to be a useful scoring system to predict the incidence of AE or future mortality in patients with ILDs, there are several limitations in the present study. First, the number of enrolled patients was still small and from a single institution. In particular, there were much fewer patients with the clinical diagnoses of CHP or UC-ILD. Several previous cohort studies on IPF, fHP, and UC-ILD have examined the usefulness and limitations of the GAP or ILD-GAP models [26–30]. It is also necessary to verify the usefulness of each ILD subtype in the ILD-GAPM model, because in the present study, unlike the IPF group, no significant difference in prognosis between the high and low monocyte ratio groups was found in the non-IPF group. Second, the majority of patients enrolled were not so severely ill that pulmonary function tests including D_Lco_ could not be tolerated, which suggests a possible source of bias in the present study, though we found that the same tendency for all enrolled patients were observed among the patients with lower D_Lco_. Third, the present study assessed the prognostic predictability of the blood monocyte ratio at baseline, but the clinical significance of changes over time was not evaluated. If circulating monocytes are considered to be a biomarker related to future fibrosis formation, it would be expected that the prognosis would be poor in patients with increasing or persistently high blood monocyte counts. This could be an important future direction of this study. Fourth, in the present study, the monocyte ratio did not correlate with clinical parameters such as KL-6 and FVC. Though blood monocytes have a novel aspect as an ILD biomarker, it seems necessary to evaluate the relevance to macrophage activation markers such as HO-1.

## Conclusions

The blood monocyte ratio could be an important predictor of 3-year ILD-related events among the patients with ILD (especially IPF). Also, the clinical usefulness of the ILD-GAPM model seems to require verification for each ILD subtype, however since the blood monocyte ratio is a very easily measurable biomarker, it is expected that the ILD-GAPM model will be widely accepted as the prognosis prediction tool especially in early-stage ILD patients in the future.

### Supplementary Information

**Additional file 1.** The statement of TRIPOD.

## Data Availability

The data used to support the findings of this study are available from the corresponding author upon request.

## Abbreviations

AE: Acute exacerbation
AIC: Akaike’s information criterion
AISI: Aggregate index of systemic inflammation
ATS/ERS/JRS/ALAT: American thoracic society/European respiratory society/Japanese respiratory society/Latin american thoracic association
AUC: Areas under the time-dependent ROC analysis
CCIS: Charlson Comorbidity Index score
CHP: Chronic hypersensitivity pneumonia
CI: Confidence intervals
C-index: The concordance index
CVD-IP: Collagen vascular disease-related interstitial pneumonia
fHP: Fibrotic HP
GAP: Gender / age / physiology
GAPM: Gender / age / physiology / blood monocyte ratio
GGO: Ground glass opacity
HO: Heme oxygenase
HRCT: High-resolution computed tomography
IIP: Idiopathic interstitial pneumonia
ILD: Interstitial lung disease
iNSIP: idiopathic NSIP
IPF: Idiopathic pulmonary fibrosis
LDH: Lactate dehydrogenase
NLR: Neutrophil-to-lymphocyte ratio
NSIP: Non-specific interstitial pneumonia
%FVC: percentage predicted forced vital capacity
%D_Lco_: percentage predicted diffusion capacity of lung for carbon monoxide
PLR: Platelet-to-lymphocyte ratio
ROC: Receiver-operating characteristic curve
SII: Systemic inflammation index
SIRI: Systemic inflammation response index
SP: Surfactant protein
UC-ILD: Unclassifiable ILD
UIP: Usual interstitial pneumonia
